# Human immune responses that reduce the transmission of *Plasmodium falciparum* in African populations

**DOI:** 10.1016/j.ijpara.2010.09.008

**Published:** 2011-03

**Authors:** Teun Bousema, Colin J. Sutherland, Thomas S. Churcher, Bert Mulder, Louis C. Gouagna, Eleanor M. Riley, Geoffrey A.T. Targett, Chris J. Drakeley

**Affiliations:** a Department of Immunology and Infection, Faculty of Infectious and Tropical Diseases, London School of Hygiene and Tropical Medicine, London, UK; b Department of Infectious Disease Epidemiology, Faculty of Medicine, Imperial College London, London, UK; c Microbiology Laboratory Twente, Enschede, The Netherlands; d Institut de Recherche pour le Développement, Marseille, France

**Keywords:** Membrane feeding, Malaria, Sexual stage immunity, Acquisition, Longevity, Dynamics, Pfs48/45, Pfs230

## Abstract

Malaria-infected individuals can develop antibodies which reduce the infectiousness of *Plasmodium* gametocytes to biting *Anopheles* mosquitoes. When ingested in a bloodmeal together with gametocytes, these antibodies reduce or prevent subsequent parasite maturation in the insect host. This transmission-blocking immunity is usually measured in human sera by testing its effect on the infectivity of gametocytes grown in vitro. Here we evaluate evidence of transmission-blocking immunity in eight studies conducted in three African countries. *Plasmodium falciparum* gametocytes isolated from each individual were fed to mosquitoes in both autologous plasma collected with the parasites, and permissive serum from non-exposed donors. Evidence of transmission reducing effects of autologous plasma was found in all countries. Experiments involving 116 Gambian children (aged 0.5–15 years) were combined to determine which factors were associated with transmission reducing immune responses. The chances of infecting at least one mosquito and the average proportion of infected mosquitoes were negatively associated with recent exposure to gametocytes and sampling late in the season. These results suggest that effective malaria transmission-reducing antibodies do not commonly circulate in African children, and that recent gametocyte carriage is required to initiate and/or boost such responses.

## Graphical abstract

**Graphical abstract:**

**Figure:**
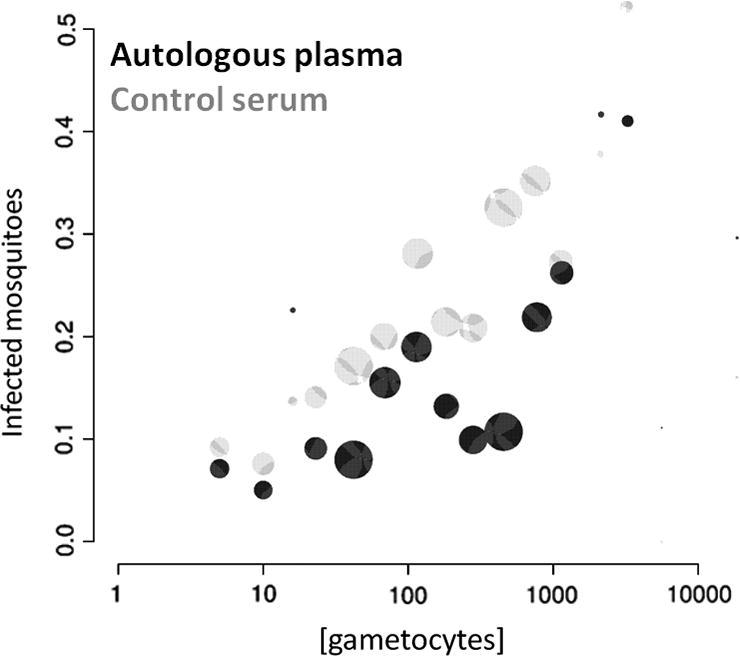

**Research highlights:**

► Gametocyte density is positively associated with mosquito infection rates. ► Autologous plasma reduces the transmission of malaria parasites. ► Transmission reducing activity is associated with indicators of recent exposure to gametocytes.

## 1 Introduction

Recent successes in malaria control (Barnes et al., 2005, 2009; Bhattarai et al., 2007; Ceesay et al., 2008; Kleinschmidt et al., 2009) have resulted in optimism about the possibility of eliminating malaria in many areas where the disease is currently endemic (Guerra et al., 2008). Transmission reducing interventions are now acknowledged as key components of malaria control and elimination efforts (Greenwood, 2008; Greenwood et al., 2008; White, 2008). The transmission of malaria depends on the presence of infectious sexual stage malaria parasites, gametocytes, in the human peripheral blood. These gametocytes do not cause clinical disease but once ingested by mosquitoes taking a blood meal, can develop into ookinetes, oocysts and ultimately sporozoites, thereby rendering the mosquito infectious to human beings. The infectiousness of gametocytes is influenced by their concentration (Jeffery and Eyles, 1955; Tchuinkam et al., 1993; Schneider et al., 2007), degree of maturity (Targett et al., 2001; Hallett et al., 2006) and by mosquito (Whitten et al., 2006) and human immune responses (Bousema et al., 2006a). The development of a human immune response to gametocytes is not surprising given that the vast majority of gametocytes are not taken up by mosquitoes but are cleared by the host immune system. There is indirect evidence that human immune responses may actively clear circulating gametocytes after recognising antigens on the gametocyte-infected erythrocyte (Baird et al., 1991; Taylor and Read, 1997; Saeed et al., 2008). A distinct human immune response may also reduce the infectiousness of gametocytes. Naturally occurring transmission reducing activity (TRA) has been associated with antibodies against antigens that are internally expressed in gametocytes but appear on the surface of gametes after gametocytes have been ingested by mosquitoes, notably Pfs48/45 and Pfs230 (Carter et al., 1990; Roeffen et al., 1995; Bousema et al., 2006a). TRA forms the basis for the development of transmission blocking vaccines (Carter et al., 2000; Pradel, 2007; Saul, 2008) that could play a key role in malaria elimination efforts (Sauerwein, 2007; Targett and Greenwood, 2008; Greenwood and Targett, 2009) in particular by removing the asymptomatic reservoir from which mosquitoes can be infected.

Two types of assays are commonly used to detect TRA: the standard membrane feeding assay (SMFA) and the direct membrane feeding assay (DMFA) (Bousema et al., 2006a). In the SMFA, cultured gametocytes are fed to *Anopheles* mosquitoes in the presence of an (endemic) test serum or plasma or non-malaria control serum (Ponnudurai et al., 1989); in the DMFA, which can be conducted in the field, blood samples from naturally infected gametocyte carriers are fed to mosquitoes in the presence of autologous plasma (AP) or control serum (CS), after a washing step (Tchuinkam et al., 1993). Advantages of the DMFA are that it uses parasite strains that are naturally circulating in the study population, gametocyte densities that are representative of the natural situation and locally caught and reared mosquitoes. The DMFA may therefore resemble the natural situation better than the SMFA. However, due to the labour intensiveness of the assay, depending on the dissection of typically 20–60 mosquitoes per experiment, studies using DMFA are often too small to reliably confirm the existence of TRA in endemic populations, let alone to explore factors associated with TRA. Consequently, several fundamental questions about the nature of TRA remain. TRA is thought to be rapidly induced (Bousema et al., 2006b) but short-lived (Bousema et al., 2006a, 2007; Drakeley et al., 2006b) but both of these assertions are yet to be confirmed in field studies. To investigate the induction, duration and efficacy of anti-gamete antibodies in natural infections, we determined the presence of TRA and associated factors in combined data from eight membrane-feeding studies conducted in The Gambia, Kenya and Cameroon.

## 2 Materials and methods

### 2.1 Field studies

Data from eight trials with naturally infected individuals from The Gambia, Kenya and Cameroon were included in the current study (Table 1). With the exception of a subset of the experiments from Cameroon, these data were not previously analysed to determine TRA. Experiments from The Gambia and Kenya involved feeds on blood samples obtained from children after anti-malarial treatment for a clinical malaria episode; samples from Cameroon were collected prior to treatment from both symptomatically and asymptomatically- infected individuals.

#### 2.1.1 Data from The Gambia

The study site, recruitment process, treatment regimes and feeding experiments have been described previously (Targett et al., 2001; Drakeley et al., 2004b; Sutherland et al., 2005). Briefly, malaria is seasonal and most transmission occurs between August and December. The transmission intensity at the time of the studies was of the order of 10–20 infective bites/person/year. Children attending the health centre at Farafenni were recruited in the transmission season. Eligible children were aged 0.5–15 years with a history of fever and *Plasmodium falciparum* asexual parasitaemia >500/μL of blood in the absence of other species of *Plasmodium*. Exclusion criteria included anaemia (packed cell volume (PCV) < 20%); signs of severe malaria; inability to take drugs orally; reported treatment with any anti-malarial within the past 2 weeks; and any evidence of chronic disease or other acute infection. After obtaining consent from parents or guardians, children were randomly assigned to different treatment regimens (Table 1). Following treatment, patients were brought to the Medical Research Council (MRC) laboratory in Farafenni for membrane-feeding experiments on days 4 (*n* = 45), 7 (*n* = 126), 10 (*n* = 6) and 14 (*n* = 9) after treatment. The study protocols were approved by both the Joint Gambia Government/MRC Ethics Committee and the London School of Hygiene and Tropical Medicine Ethics Committee.

#### 2.1.2 Data from Kenya

The study in Kenya was conducted in Mbita, western Kenya, on the shores of Lake Victoria. Symptomatic children aged 6 months-10 years with uncomplicated malaria and a *P. falciparum* mono-infection with a density of at least 1000 parasites/μL were recruited. Exclusion criteria were similar to those described for the studies in The Gambia; anaemia in this case was defined as haemoglobin concentration lower than 5 g/dL. Children were randomized to receive either artemether–lumefantrine (AL) or dihydroartemisinin–piperaquine. Seven days after enrolment, individuals aged 2–10 years with and without microscopically confirmed gametocytes were recruited for membrane-feeding experiments; however, the current analyses were restricted to microscopically confirmed gametocyte carriers. The study protocol received ethical approval from the Ethical Review Committee of the Kenya Medical Research Institute and the Ethics Committee of the London School of Hygiene and Tropical Medicine The trial was registered online at <http://clinicaltrials.gov/ct2/show/NCT00868465>.

#### 2.1.3 Data from Cameroon

Two separate studies were conducted in Cameroon in 1995–1998 (Cameroon 1 (Bonnet et al., 2000; Gouagna et al., 2004)) and in 1995–1996 (Cameroon 2 (Mulder et al., 1999)). In the first study, gametocyte carriers (aged 4–38 years) were recruited during community-wide cross-sectional surveys in the district of Mengang, where annual malaria transmission intensity is around 100 infective bites/person/year (Bonnet et al., 2003). In the second study, 55 gametocyte carriers (aged 1–63 years) were recruited among patients of the Messa dispensary in an urban quarter of Yaoundé and exposed to a transmission intensity of ∼34 infectious bites per person per year (van der Kolk et al., 2003). Of these, 5.5% (3/55) presented with a temperature ⩾37.5 °C (Mulder et al., 1999). In both studies, individuals with gametocytes by microscopy were selected for membrane-feeding experiments. Individuals with asexual parasites received anti-malarial treatment following national guidelines after sampling for membrane feeds was completed. The projects were approved by the National Ethical Clearance Committee for Cameroon.

### 2.2 Membrane feeding and mosquito dissection

Venous blood samples (2–4 ml) were obtained from children whose parent or guardian had given specific consent for the procedure. Venous blood in citrate–phosphate dextrose (The Gambia) or heparin (Cameroon, Kenya) was centrifuged, and the plasma was removed. After being washed, the red blood cell pellet was split into two aliquots of 300–500 μL each. These were resuspended to a PCV of 33% in, respectively, the original AP and in pooled AB serum from European donors with no history of malaria exposure (CS). Each suspension then was fed to 50–100 3–5 day old female *Anopheles gambiae* sensu stricto mosquitoes. In studies from The Gambia, the next generation progeny of wild-caught gravid female mosquitoes were used; locally reared laboratory strain mosquitoes that were adapted to feeding on a membrane feeder were used in Cameroon (Tchuinkam et al., 1993) and Kenya (Bousema et al., 2006c). In all studies, starved mosquitoes were allowed to feed for 15–30 min via an artificial membrane attached to a water-jacketed glass feeder maintained at 37 °C. After feeding, blood-fed mosquitoes were kept at 26–28 °C with permanent access to a 10% sucrose solution without further blood meals. Mosquito midguts were dissected out 7–8 days later in PBS (The Gambia) or 2% (Cameroon, The Gambia) or 0.5% mercurochrome (Kenya) in distiled water; the number of oocysts – a developmental stage of the parasite found on the insect midgut – was recorded.

### 2.3 Data analysis

Data were entered using Epi-Info or MS-Access and analysed using Stata version 11 (Stata Corporation, Texas, USA). Analyses were restricted to experiments on microscopically confirmed gametocyte carriers that had at least 10 mosquitoes dissected and resulted in at least one infected mosquito in the CS and/or AP experiment. The latter criterion was invoked to rule out technical problems with the DMFA. The relationship between gametocyte density and mosquito infection rates was visualised for all data combined by grouping mosquito feeding experiments according to the density of gametocytes in the blood ingested (into 20 evenly spaced groups on the log-scale). A Bland–Altman (difference) plot was created to visualise the difference between the proportion of mosquitoes that was infected after feeding on a blood sample with CS or AP. Oocyst counts were highly over-dispersed (mean = 2.19; S.D. = 11.51) and were presented in categories: 0, 1–2, 3–5, 6–15, 16–50 and >50 oocysts.

The studies in The Gambia recruited individuals from a wide age range (0.5–15 years) who were treated with artemisinin-combination therapy (ACT) and non-ACT treatment, recruited at the start and through the transmission season and of whom a proportion presented with gametocytes at enrolment, i.e. 4–14 days before membrane feeding. We therefore used the combined datasets from The Gambia to determine factors associated with TRA. The influence of host factors was examined in two ways. Firstly, the prevalence of infectiousness among donors, defined as the proportion of individuals that provided a gametocyte-positive blood sample which resulted in at least one infected mosquito, was compared between CS and AP feeds for: different age-groups (<5 compared with ⩾5 years); season of enrolment (peak compared with start of transmission season); treatment (non-ACT compared with ACT); history of microscopic gametocytaemia (gametocyte-free versus gametocytes present at enrolment); gametocyte density at the day of membrane feeding above or below the median value (50 gametocytes/μL). Different feeding days were combined. The McNemar test was used to test for a difference between paired CS and AP experiments. Subsequently, the proportion of infected mosquitoes was compared between CS and AP feeds for the same categories of participants. A multilevel logistic regression model was used for this purpose using a multilevel generalised linear model (GLLAMM, Stata version 11; Stata Corporation, Texas, USA). This model incorporated clustering per patient and random effects to account for differences between studies. Because mosquito infection rates were strongly associated with gametocyte density, all analyses except those directly testing the influence of having a high gametocyte density (⩾50 gametocytes/μL) at the time of membrane feeding were adjusted for log (ln) transformed gametocyte density. The GLLAMM model was also used for multivariate analyses where experiments with CS were used as reference category. Interaction terms were included in the model; variables were selected for the multivariate model if *P* < 0.05 in the univariate analyses and retained in the model if *P* < 0.10.

## 3 Results

### 3.1 The association between gametocyte density and mosquito infection rates

**Fig. 1:**
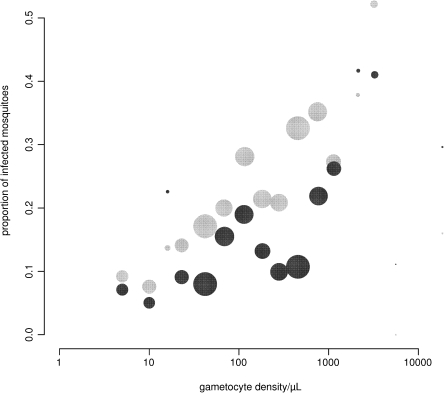
The relationship between gametocyte density by microscopy and the proportion of infected mosquitoes. Light grey circles indicate the proportion of infected mosquitoes after feeding on blood samples with control serum (CS); dark grey circles indicate autologous plasma (AP). The sizes of the circles are proportional to the number of mosquitoes dissected for a given range of gametocyte densities. The dataset combines observations from studies in The Gambia (1998–2002; *n* = 106), Kenya (2009; *n* = 11) and Cameroon (1995–1998; *n* = 74).

**Fig. 2:**
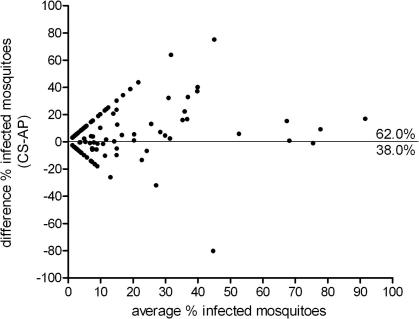
Bland–Altman (difference) plot comparing paired experiments with control serum (CS) and autologous plasma (AP). Each dot represents a paired CS-AP experiment. The mean proportion of infected mosquitoes is given on the *X*-axis and the difference between AP and CS experiments on the *Y*-axis. A positive value indicates a higher mosquito infection rate for CS feeds. In 68.2% (150/220) of the paired experiments the mosquito infection rate was higher in the CS experiment compared with the AP experiment (positive values); in 29.5% (65/220) of the experiments the infection rate was higher in the AP experiment (negative values); in 2.3% (5/220) of the experiments the proportion of infected mosquitoes was identical for CS and AP feeds.

There was a positive association between gametocyte density and the proportion of infected mosquitoes in CS and AP experiments when all studies were combined (Fig. 1). Mosquito infection rates were consistently higher in CS experiments compared with AP experiments (Figs. 1 and 2). The difference between mosquito infection rates in paired CS-AP experiments was plotted against the average proportion of infected mosquitoes (Fig. 2). This Bland–Altman plot indicated that in 68.2% of the paired experiments the proportion of infected mosquitoes in the CS experiment was higher than that in the AP experiment (*P* = 0.006).

**Fig. 3:**
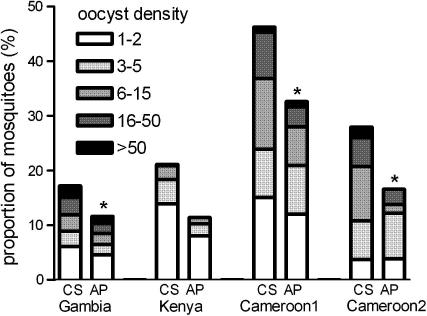
Oocyst burdens in mosquitoes after feeding on blood samples with control serum (CS) or autologous plasma (AP) in different experiments. Bars indicate the proportion of mosquitoes with the indicated oocyst burden after feeding on blood samples with control serum (CS) or autologous plasma (AP) in studies in The Gambia (1998–2002), Kenya (2009), Cameroon 1 (1997) and Cameroon 2 (1995). The asterisk indicates a statistically significant difference between CS and AP experiments in the proportion of mosquitoes with ⩾1 oocyst.

The proportion of infected mosquitoes was statistically significantly higher in CS experiments compared with AP experiments for The Gambia (Fig. 3, *P* < 0.001), Cameroon 1 (*P* = 0.03) and Cameroon 2 (*P* = 0.004), after adjustment for the correlation between observations from the same individual and study-year (only applicable for The Gambia). The increased proportion of infected mosquitoes in the CS experiments compared with the AP experiments was not statistically significant for the trial in Kenya (*P* = 0.29). Among infected mosquitoes, there was little evidence for a difference in oocyst burden between CS and AP feeds. There was no difference in intensity of infection (oocyst counts among infected mosquitoes) between CS and AP feeds in the combined Gambia data (*P* = 0.92), the Kenya data (*P* = 0.11) and the first trial in Cameroon (*P* = 0.12), after adjusting for correlation between observations from the same individual and, where applicable, study-year. Oocyst counts in infected mosquitoes were higher in CS compared with AP feeds in the second trial in Cameroon (*P* = 0.01).

### 3.2 Factors associated with TRA

The dataset from the combined Gambian trials was the largest dataset and provided most details of human host factors. Because these trials showed no evidence for differing oocyst counts among infected mosquitoes between CS and AP feeds, the analyses were done on the prevalence of infectiousness (i.e. the proportion of samples infecting at least one mosquito) and the proportion of infected mosquitoes. The proportion of individual donor samples that infected at least one mosquito was higher after serum replacement, although at borderline significance (*P* = 0.05, Table 2). This difference was more pronounced when the proportion of infected mosquitoes was considered (*P* < 0.001). A higher infectivity in CS compared with AP feeds was found for samples from children older than 5 years of age, samples from individuals who presented with gametocytes at enrolment and samples from individuals who were sampled at the end of the transmission season. The proportion of infected mosquitoes increased after serum replacement for individuals with low and high gametocyte densities but the effect of serum on transmissibility was more pronounced in those with higher gametocyte densities. The type of treatment (ACT versus non-ACT) did not influence the transmission-reducing capacity of serum.

To determine independent predictors of TRA, a multivariate model was built with CS experiments as the reference group (Table 3). This model adjusted estimates for gametocyte density at the time of feeding and correlations between observations from the same individual, and incorporated a random effect for study-year. Factors associated with TRA in the univariate analysis were identical to those in Table 2: gametocytes present at the preceding enrolment visit, older age and sampling late in the season. In the multivariate model, however, only the presence of gametocytes at enrolment and sampling late in the season were significantly associated with lower mosquito infection rates after adjustment for gametocyte density, correlations between observations from the same individual and study year.

## 4 Discussion

In this study, we describe the prevalence of transmission reducing immune responses in naturally infected individuals from three malaria endemic countries. All studies showed evidence for TRA that was associated with several indicators of increased recent exposure to gametocytes.

When combining successful membrane-feeding experiments of 201 naturally infected individuals, we observed a clear association between mosquito infection rates and gametocyte density. Although this association was evident for both experiments with permissive CS from non-exposed donors and experiments using AP, mosquito infection rates were consistently higher in CS experiments. Replacing AP by CS resulted in significantly higher infection rates in two-thirds of all membrane-feeding experiments. The higher infectivity in CS feeds was well captured by the prevalence scale (i.e. the presence or absence of oocysts) and there was little to be gained by analysis of oocyst densities in infected mosquitoes in most of the datasets. In univariate analyses, higher gametocyte densities at the time of membrane feeding, gametocyte carriage during the week prior to the day of membrane feeding, older age of children and sampling later in the transmission season were all associated with TRA, defined as either a higher likelihood of transmission-success in paired CS-AP experiments or a higher proportion of infected mosquitoes in CS experiments. A multivariate model indicated that the presence of gametocytes during the week prior to the experiment and sampling late in the transmission season were independently associated with increased TRA. Increased TRA in children that carried gametocytes during the week prior to the feeding experiment suggests that recent exposure to gametocytes is associated with TRA. A rapid acquisition of antibody responses to Pfs48/45 and Pfs230, both associated with naturally acquired TRA (Bousema et al., 2006a), was previously shown in individuals newly exposed to malaria (Ong et al., 1990; Bousema et al., 2006b). It is therefore plausible that transmission-reducing antibodies were acquired or boosted in response to recent exposure to gametocyte antigens. Some exposure to gametocyte antigens will have remained undetected by relying on microscopy that may underestimate gametocyte carriage considerably (Ali et al., 2006; Bousema et al., 2006c; Schneider et al., 2006; Shekalaghe et al., 2007; Mens et al., 2008). It is plausible that some of the individuals who developed gametocytes after treatment had sub-patent gametocytes at enrolment (Ali et al., 2006; Schneider et al., 2006). These individuals, however, were less likely to show transmission reducing immune responses at the time of feeding than those who had microscopically detectable gametocytes at enrolment. This suggests that exposure to relatively high density (i.e. microscopically detectable) gametocytes several days before the experiment is needed to elicit functional TRA. This observation is in line with the hypothesis that transmission reducing immunity is rapidly induced and depends on recent exposure to gametocytes (Bousema et al., 2006a). A similar biological mechanism could be responsible for the observed increase in TRA towards the end of the transmission season. Exposure to gametocytes shows considerable seasonal fluctuation with a higher prevalence and density of gametocyte carriage in the transmission season compared with the dry season (Drakeley et al., 2006a; Ouedraogo et al., 2008). The proportion of children who have been exposed to gametocytes in the weeks before the membrane-feeding experiments will therefore be highest towards the end of the transmission season, resulting in higher levels of TRA. These findings also indirectly support the short-lived nature of transmission reducing immune responses. If cumulative life-long exposure to gametocytes was key to the acquisition and maintenance of transmission reducing immune responses, as observed for pre-erythrocytic and blood-stage immune responses (Del Giudice et al., 1990; Wipasa et al., 2002), one would expect age to be the dominant factor predicting TRA. An effect of age on infectiousness that is independent of gametocyte density was found in some (Githeko et al., 1992; Toure et al., 1998; Schneider et al., 2007) but not all (Graves et al., 1988a; Tchuinkam et al., 1993; Drakeley et al., 2000) studies. Our experiments from The Gambia only included children with a median age of 4 years (range 0.5–15 years) and we therefore cannot extrapolate our findings to adults, who appear to have a markedly lower exposure to gametocytes than children (Drakeley et al., 2006a). In multivariate models, age lost its influence on TRA after adjustment for recent exposure to gametocytes. This suggests the association between age and transmission reducing immune responses is complex. Age could be an indicator of better developed clinical immune responses that change malaria disease progression and treatment seeking behaviour. We hypothesise that in our population of children, older age allowed infections to remain asymptomatic for a longer period, thereby postponing treatment seeking (von Seidlein et al., 2002; Mota et al., 2009) and allowing for a longer period of potential gametocyte production. This would again indicate an influence of higher recent exposure to gametocytes in older children.

When considering our individually paired AP–CS experiments, we found evidence for an increased transmission in AP compared with CS experiments in 3.4% of the experiments (4/116; data not shown). The phenomenon of transmission enhancement has been reported in other field studies (Graves et al., 1988b; Premawansa et al., 1994) and may be a real biological phenomenon rather than an artefact resulting from variation in membrane feeding assays (van der Kolk et al., 2006). The biological mechanism is unclear. In some studies (Peiris et al., 1988; Gamage-Mendis et al., 1992; Healer et al., 1999) transmission enhancement was linked to the presence of very low concentrations of anti-gamete antibodies but this was not confirmed in the largest study on transmission enhancement in *P. falciparum* (van der Kolk et al., 2006). The importance of this phenomenon at a population level will require further study and our limited number of samples showing enhancement did not allow us to determine associated factors.

Our findings illustrate some of the difficulties encountered when doing transmission experiments under field conditions. Mosquito infection rates differed between study sites (Stepniewska et al., 2008) and the association between gametocyte densities and mosquito infection rates can be highly variable (Graves, 1980; Carter and Graves, 1988; Sinden et al., 1996; Boudin et al., 2004; Coleman et al., 2004; Schneider et al., 2007; Ouedraogo et al., 2009). Even when cultured gametocytes are used under highly standardised laboratory conditions, the proportion of infected mosquitoes and the number of oocysts resulting from a given gametocyte density can vary considerably (van der Kolk et al., 2004). This variation is largest at low gametocyte concentrations (van der Kolk et al., 2004) and is partly a consequence of the narrow age range over which gametocytes are infectious (Lensen et al., 1999; Hallett et al., 2006). The type of anticoagulant used may affect oocyst development (Ponnudurai et al., 1989; Solarte et al., 2007), an effect that has been best described for EDTA, which may reduce oocyst numbers (Solarte et al., 2007). Such an effect will not have influenced our conclusions since our analyses were based on pair-wise comparisons of samples from the same individual (i.e. with the same anticoagulant) or restricted to data from the Gambia where only citrate phosphate dextrose was used. Similar to variation in the highly standardised SMFA, we expect there will be considerable variation in TRA measured by DMFA (Mulder et al., 1999; van der Kolk et al., 2004). This makes it complicated to draw firm conclusions from individual cross-sectional observations on TRA. Longitudinal studies are therefore needed to test the causality of our findings. In future longitudinal studies the acquisition rate and longevity of TRA should be determined in repeated membrane feeding assays on naturally infected individuals.

The current findings confirm the presence of TRA in naturally-exposed populations and provide biologically plausible support for the hypothesis that TRA depends on recent exposure to gametocytes. It is well established that transmission can be reduced by antibodies to the gametocyte/gamete antigens Pfs 48/45, Pfs 230 and PfHAP2 (Sauerwein, 2007; Targett and Greenwood, 2008) and the ELISA titer of antibodies against these antigens is related to the level of TRA (Roeffen et al., 1996; Drakeley et al., 2004a; Bousema et al., in press). Further dissection of the specific contribution to TRA of these antibodies, and of other immune responses, is required. The data presented here strongly suggest that recent gametocyte exposure is a requirement for effective TRA. Thus vaccines designed to block transmission by mimicking natural TRA will have to overcome this requirement for boosting by antigen exposure, if they are to be effective tools for reducing or interrupting transmission of *P. falciparum.*

## Figures and Tables

**Table 1:** Summary membrane-feeding experiments on microscopically confirmed gametocyte carriers with paired Autologous Plasma (AP) and Control Serum (CS) observations. Only gametocyte carriers who had a minimum of 10 mosquitoes dissected in both AP and CS feeds were included in the analyses.

Country	Year	Timing of membrane feed experiments	Median gametocyte density (IQR)	Number of combined AP-CS feeds (total mosquitoes CS;AP)	% successful feeds (*n*/*N*)^a^
Farafenni, The Gambia	1998 (Targett et al., 2001)	Four (*n* = 45) or 7 days (*n* = 10) after treatment with CQ, SP, SP + AS1 or SP + AS3	120 (48–376)	55 (1439;1272)	56.4 (31/55)
Farafenni, The Gambia	1999 (Targett et al., 2001)	Seven days after treatment with SP or SP + AS3	20 (10–220)	33(701;715)	100.0 (33/33)
Farafenni, The Gambia	2000 (Drakeley et al., 2004)	Seven days after treatment with CQ or CQ + AS3	50 (10–245)	38 (652;622)	73.7 (28/38)
Farafenni, The Gambia	2001 (Hallett et al., 2006)	Seven (*n* *= *27), 10 (*n* = 6) or 14 (*n* = 8) days after treatment with CQ, SP or CQ + SP	100 (35–615)	41(782;750)	48.8 (20/41)
Farafenni, The Gambia	2002 (Sutherland et al., 2005)	Seven days after treatment with CQ + SP or AL	53 (20–160)	19(368;373)	21.1 (4/19)
**Farafenni, The Gambia**	**1998–2002**	**After treatment with anti-malarials**	**65(20–320)**	**186 (3942; 3732)**	**62.4 (116/186)**
**Mbita, Kenya**	**2009**	**After treatment with AL or DP**	**200 (120–440)**	**12 (360;360)**	**91.6 (11/12)**

**Mengang, Cameroon 1**	**1997 (****Bonnet et al., 2000; Gouagna et al., 2004****)**	**Prior to treatment**	**192 (80–608)**	**19 (519;540)**	**100 (19/19)**
**Yaoundé, Cameroon 2**	**1995 (****Mulder et al., 1999****)**	**Prior to treatment**	**296 (88–536)**	**55 (1465;1086)**	**100 (55/55)**

Bold lines indicate the summary figures per country. IQR, interquartile range (25th–75th percentile); CQ, chloroquine; SP, sulphadoxine-pyrimethamine; AS1, one dose of artesunate given together with SP; AS3, three doses of artesunate; AL, artemether-lumefantrine; DP, dihydroartemisinin-piperaquine.

a Defined as at least one infected mosquito in feeding experiment using AP and CS.

**Table 2:** Factors associated with transmission reducing activity (TRA) in The Gambian dataset.

Variable	Prevalence of infectiousness	*P*-value^a^	Proportion infected mosquitoes	OR, *P*-value^b^
All data	AP: 63.8 (74/116) CS: 77.6 (90/116)	*p* = 0.05	AP: 11.6 (278/2391) CS: 17.2 (433/2518)	0.60 (0.50–0.72) *P *< 0.001

*Gametocyte density at feeding*				
<50 gametocytes/μL	AP: 62.2 (31/50) CS: 70.0 (35/50	*p* = 0.49	AP: 7.2 (76/1049) CS: 10.2 (112/1101	0.72 (0.52–0.99) *P = *0.045
⩾50 gametocytes/μL	AP: 65.2 (43/66) CS: 83.3 (66/66)	*p* = 0.04	AP: 15.0 (201/1341) CS: 22.7 (321/1417)	0.54 (0.43–0.68) *P < *0.001

*Gametocyte prevalence at presentation*				
Yes	AP: 56.1 (23/41) CS: 87.8 (36/41)	*p* = 0.007	AP: 10.8 (88/818) CS: 22.4 (183/817)	0.36 (0.26–0.49) *P *< 0.001
No	AP: 64.7 (44/68) CS: 75.0 (51/68)	*p* = 0.27	AP: 11.6 (168/1449) CS: 13.2 (203/1541)	0.87 (0.68–1.11) *P *= 0.26

*Age*				
Under 5 years	AP: 65.4 (34/52) CS: 67.3 (35/52)	*p* = 0.87	AP: 9.5 (101/1061) CS: 10.5 (116/1101)	0.84 (0.62–1.14) *P *= 0.27
Over 5 years	AP: 60.0 (33/55) CS: 89.1 (49/55)	*p* = 0.003	AP: 12.5 (140/1123) CS: 19.5 (232/1193)	0.55 (0.43–0.71) *P *< 0.001

*Drug*				
Non-ACT	AP: 63.6 (49/77) CS: 77.9 (60/77)	*p* = 0.10	AP: 11.3 (180/1595) CS: 16.7 (270/1616)	0.59 (0.47–0.74) *P *< 0.001
ACT	AP: 64.1 (25/39) CS: 76.9 (30/39)	*p* = 0.30	AP: 12.3 (98/796) CS: 18.1 (163/902)	0.62 (0.45–0.86) *P *= 0.004

*Season*				
Early	AP: 69.6 (32/46) CS: 73.9 (34/46)	*p* = 0.69	AP: 11.7 (101/865) CS: 11.8 (105/888)	0.99 (0.72–1.38) *P *= 0.97
Late	AP: 57.1 (40/70) CS: 82.9 (58/70)	*p*<0.001	AP: 11.6 (177/1526) CS: 20.1 (328/1630)	0.47 (0.37–0.59) *P *< 0.001

a By McNemar test for paired control serum-autologous plasma (CS–AP) observations.

b By Generalised Linear Latent and Mixed Models (GLAMM), adjusting for gametocyte density at the time of feeding (except for the variable ‘gametocyte density’) and correlations between observations from the same individual and study-year. OR, odds ratio.

**Table 3:** Independent predictors of mosquito infection prevalence in the Gambian dataset. Results of Generalised Linear Latent and Mixed Models (GLAMM) on the proportion of infected mosquitoes. Estimates were adjusted for gametocyte density, the correlation between observations from the same individuals and a random effect was added for study year. Variables were added to the model if *P < *0.05 in the univariate model (adjusting for gametocyte density) and retained in the model if *P < *0.05 in the multivariate model through backward elimination of non-significant variables.

	Univariate OR (95% CI)	*P*-value	Multivariate OR (95% CI)	*P*-value
Control serum	1.0 (ref)		1.0 (ref)	

*Autologous plasma*				
Gametocytes at enrolment	0.36 (0.26–0.49)	<0.001	0.52 (0.37–0.74)	<0.001
Over 5 years of age	0.58 (0.45–0.75)	<0.001	–	
Late in the season	0.49 (0.39–0.61)	<0.001	0.62 (0.48–0.80)	<0.001
Gametocyte density (ln)/μL	1.34 (1.17–1.53)	<0.001	1.40 (1.20–1.62)	<0.001

OR, odds ratio; CI, confidence interval; ref, reference category.
